# Supplementary material for: Bilayer Forming Phospholipids as Targets for Cancer Therapy
Source: Int J Mol Sci. 2022 May 9;23(9):5266. doi: 10.3390/ijms23095266 (PMC9100777; doi:10.3390/ijms23095266)
Supplement: Supplementary file 1 [file ijms-23-05266-s001.zip › ijms-1668931-supplementary.pdf]

Supplementary Material

Table S1. Primers used for PCR experiments.

Gene	Forward	Reverse	Product length (bp)
PCYT2-201	GCACAGTGGAGAGGCCGG	TGGAGCCAGGCGCTGGGT	273
PCYT2-202 (PCYT2α)	GGAGATGTCCTCTGAG-TACCG	GGCACCAGCCACATA-GATGAC	226
PCYT2-203 (PCYT2β)	Same as *PCYT2α*	Same as *PCYT2α*	172
PCYT2-205	GCTATGACATGGTG-CATTACG	CTCCCAGCCTGCTTTACTTC	313
PCYT2-207	Same as *PCYT2-205*	GGTAGTTCTTCCCCTT-GTAGTG	876
PCYT2-208	AGTCTCCGGGCTGAGCTA	CTTGAAGTGACTTAG-GAGCTC	180
PCYT2-209	AGATCTGGAGCCACGGCC	CCATTTGATGGCCTGCAC	229
PCYT2-210	GTTCTGGGTGGCGTCCTC	CAGTGGTGGGGAGCAGGT	400
PCYT2-211	GAGGTCAATCAC-TACAAGGGG	CTCCTTGGCTTCCTTCTTCTG	732
PCYT2-212	CAAAGCCCATCACAGCAG	GTATGGGTCGGAGCCATC	418
PCYT2-213 (PCYT2γ)	Same as *PCYT2α*	Same as *PCYT2α*	416
PCYT2-214	GTACAGAGAATGCAAGCGC	CTTGAAGTGACTTAG-GAGCTC	457
PCYT2-216	GTCACTGCCTTCTTGCTC	CCTCATGGGAAGAAGTCG	215
PCYT2-217	CACCTCGTTCCCTCTGTTC	CTCTGAGCAGCTTTGCTGG	667
GAPDH	CTTCAAGGTGAGGCTCC	TTCTCTTGGGCTCCTGGT	450
